# Geometric and dosimetric impact of anatomical changes for MR‐only radiation therapy for the prostate

**DOI:** 10.1002/acm2.12551

**Published:** 2019-03-01

**Authors:** Siamak P. Nejad‐Davarani, Parag Sevak, Michael Moncion, Kimberly Garbarino, Steffen Weiss, Joshua Kim, Lonni Schultz, Mohamed A. Elshaikh, Steffen Renisch, Carri Glide‐Hurst

**Affiliations:** ^1^ Department of Radiation Oncology Henry Ford Cancer Institute Detroit MI USA; ^2^ The Cancer Center Columbus Regional Health Columbus IN USA; ^3^ Radiation Oncology Department St. Jude Children's Research Hospital Memphis TN USA; ^4^ Department of Digital Imaging Philips Research Laboratories Hamburg Germany; ^5^ Department of Public Health Sciences Henry Ford Health System Detroit MI USA

**Keywords:** bladder filling, dose calculation, synthetic CT, transient anatomies

## Abstract

**Purpose:**

With the move towards magnetic resonance imaging (MRI) as a primary treatment planning modality option for men with prostate cancer, it becomes critical to quantify the potential uncertainties introduced for MR‐only planning. This work characterized geometric and dosimetric intra‐fractional changes between the prostate, seminal vesicles (SVs), and organs at risk (OARs) in response to bladder filling conditions.

**Materials and methods:**

T2‐weighted and mDixon sequences (3–4 time points/subject, at 1, 1.5 and 3.0 T with totally 34 evaluable time points) were acquired in nine subjects using a fixed bladder filling protocol (bladder void, 20 oz water consumed pre‐imaging, 10 oz mid‐session). Using mDixon images, Magnetic Resonance for Calculating Attenuation (MR‐CAT) synthetic computed tomography (CT) images were generated by classifying voxels as muscle, adipose, spongy, and compact bone and by assignment of bulk Hounsfield Unit values. Organs including the prostate, SVs, bladder, and rectum were delineated on the T2 images at each time point by one physician. The displacement of the prostate and SVs was assessed based on the shift of the center of mass of the delineated organs from the reference state (fullest bladder). Changes in dose plans at different bladder states were assessed based on volumetric modulated arc radiotherapy (VMAT) plans generated for the reference state.

**Results:**

Bladder volume reduction of 70 ± 14% from the final to initial time point (relative to the final volume) was observed in the subject population. In the empty bladder condition, the dose delivered to 95% of the planning target volume (PTV) (D95%) reduced significantly for all cases (11.53 ± 6.00%) likely due to anterior shifts of prostate/SVs relative to full bladder conditions. D15% to the bladder increased consistently in all subjects (42.27 ± 40.52%). Changes in D15% to the rectum were patient‐specific, ranging from −23.93% to 22.28% (−0.76 ± 15.30%).

**Conclusions:**

Variations in the bladder and rectal volume can significantly dislocate the prostate and OARs, which can negatively impact the dose delivered to these organs. This warrants proper preparation of patients during treatment and imaging sessions, especially when imaging required longer scan times such as MR protocols.

## 1 INTRODUCTION

Prostate cancer is the most common type of cancer in men, with over 160 000 cases reported in 2017 in the United States.^1^ The current treatment‐planning workflow involves using computed tomography simulation (CT‐SIM) as the primary planning modality. However, magnetic resonance imaging (MRI) has been shown to show superior accuracy to CT for identifying the prostate gland, the prostatic apex, and areas of high tumor burden.^2^, ^3^, ^4^, ^5^, ^6^ By performing an MRI to CT rigid registration, the prostate can be delineated on MRI and then transferred to CT for subsequent planning. This co‐registration may introduce uncertainties of 2–3 mm for prostate cancer.^7^, ^8^, ^9^ Recently, as a means to circumvent this uncertainty and streamline the clinical workflow, MR‐only planning has emerged in the clinic. For the male pelvis, two MR‐only packages are currently clinically available for prostate cancer treatment planning with synthetic CTs [synCTs, or CTs generated from MRI input(s)]. One FDA‐approved software package, the Philips Magnetic Resonance for Calculating Attenuation (MR‐CAT), is based on a dual echo three‐dimensional (3D) mDixon fast field echo sequence with synCTs generated on the scanner.^10^, ^11^ In a recent study by Farjam et al.,^12^ pelvic MR‐CAT images of 23 patients with prostate cancer underwent deformable registration to the planning CT images and found good overall agreement over the entire pelvis volume [mean absolute error (MAE) values of 65 ± 5 HU] with even smaller difference observed in the fat and muscle (~40 HU) across all subjects. However, it is not currently known how synthetic CT generation performs over a variety of internal conditions nor has the dosimetric impact of this been characterized. The other MR‐only package (Spectronic's MriPlanner) is regulatory approved (CE‐marked), and creates synCT images based on a statistical decomposition algorithm (SDA) from a single T2‐weighted dataset.^13^ Comparison of the synCT and CT‐based dose plans for prostate showed <1% difference in the mean absorbed dose to the PTV for the MR‐CAT^14^ and 0.0 ± 0.2% for the SDA methods.^13^

It has been shown that substantial variations in the bladder volume occur during the course of treatment.^15^, ^16^ Importantly, the variations in the bladder filling adversely impact the dose delivered to the prostate over a standard course of radiotherapy for the prostate.^17^, ^18^ Huang et al.^19^ used daily cone‐beam computer tomography (CBCT) images to measure target/organ volumes and dosimetric differences in 28 prostate cancer patients and found mean percentage volume differences of 44% within the bladder volumes in the treatment plan which led to percentage dose difference of 2 ± 2% in the prostate.

As MRI emerges as a primary treatment planning modality option for prostate cancer,^20^ it becomes important to quantify the potential uncertainties introduced in an MR‐only workflow due to variable physiological status that may confound accurate dosimetry and high‐precision radiation therapy. One of the main issues that arises with MRI is the longer scanning times as compared to CT, which may lead to higher variations in the bladder and rectal volumes. It is currently unknown how robust MR‐only treatment planning is to internal anatomy changes nor how the dosimetry may be impacted. This work characterizes the temporal, spatial, and dosimetric intra‐fractional changes between the prostate, seminal vesicles (SVs), and other organs at risk (OARs) in response to bladder filling conditions for MR‐only prostate cancer radiation therapy planning.

## 2 MATERIALS AND METHODS

### 2.A Subjects and bladder filling protocol

**Figure 1:**
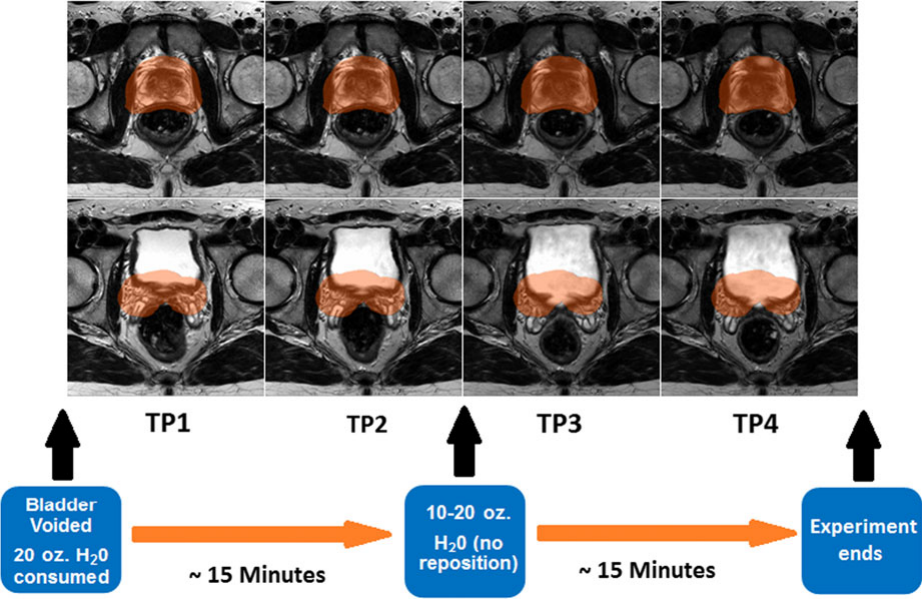
Bladder filling protocol. The highlighted planning target volume (PTV) can be seen based on the delineation of the prostate at the last time point. Effects of changes in the bladder volume can be seen in earlier time points where the shift of the prostate leads the PTV contour to overlap with the rectum as the bladder gets smaller in volume.

Nine healthy male volunteers (Age: 43 ± 10.1 yr (range: 25–61 yr); Weight: 78.8 ± 9.6 kg) were recruited and consented to being scanned at one of the three different magnetic field strengths (1.0, 1.5 or 3.0 T). Three volunteers were scanned using a large, rigid 8‐element phased array coil on a 1.0 T Panorama High Field Open Magnetic Resonance System (Philips Medical Systems, Cleveland, OH) equipped with flat table‐top (Civco, Orange City, IA) and external laser system as described previously.^21^ Three other healthy male volunteers were scanned at 1.5 T (Philips Achieva with 32‐element torso coil), and the last three on a 3.0 T (Philips Ingenia with integrated posterior 32‐element coil and anterior array) scanner with standard concave table‐tops. Pelvic MR images were acquired according to a bladder filling protocol with the patient in a supine position (Fig. 1). Prior to imaging, each subject voided their bladder and consumed 20 oz of water. The first acquisition represented an empty bladder state. An additional 10–20 oz of water was consumed without subject repositioning before 1–2 intermediate states were acquired, and a final full bladder acquisition was performed. Due to longer scan times at low magnetic fields for 1 T experiments, imaging was possible at only three time points during the ~45 min imaging session; but at 1.5 and 3 T, shorter acquisition times allowed image acquisition at three to four time points for each subject. Overall, a total of 34 evaluable time points were analyzed for the entire cohort.

### 2.B Imaging protocol

T2‐weighted turbo spin echo images were acquired since it is the most commonly used image for delineation of organs in the pelvis.^22^ The imaging protocol also consisted of dual echo 3D FFE (Fast Field Echo) mDIXON sequences^11^ which were optimized for acquisition at each field strength (3T: TR/TE1/TE2 = 3.83/1.23/2.4 ms, Voxel Size = 1.45 × 1.45 × 0.23 mm, BW = 1072 Hz; 1.5 T: TR/TE1/TE2 = 6/1.78/4 ms, Voxel Size = 1.45 × 1.45 × 0.28 mm, BW=541 Hz; 1 T: TR/TE1/TE2 = 15.87/6.9/13.81 ms, Voxel Size = 1.41 × 1.41 × 0.74 mm, BW = 975 Hz). The mDixon scan is designed to yield high‐geometric accuracy by using short echo times and high bandwidth. The advantage of using the two echoes in the mDIXON approach is to allow water, fat, and in‐phase images to be derived within the same acquisition by using the frequency shift of the fat and water protons.^11^ These images are inputted into the MR‐CAT software to produce the Synthetic CT image used for treatment planning.^10^ Briefly, MR‐CAT automatically segments the external anatomy from background air using the water and in‐phase images. Next, a model‐based segmentation method is used to segment bone from the external contour based on training datasets.^11^ Soft tissue is defined as voxels within the body volume and outside the segmented bone.^10^, ^11^, ^23^ An intensity‐based classification is then used to segment adipose and muscle within the soft tissue using the water and fat images. Finally, the bone voxels are divided into compact and spongy bone based on the voxel intensity of the in‐phase image. In summary, MR‐CAT categorizes the contents of the MR images into five classes (air, fat, water‐rich tissue, spongy bone, and compact bone) and assigns to each voxel a bulk Hounsfield Unit value based on its classification, and the final synCT image is generated for treatment planning.^11^ One current limitation of MR‐CAT is that it does not account for rectal gas in the image classification. To fully elucidate the dosimetric impact of bladder and rectal status changes, the intestinal gas with each rectal contour was automatically thresholded and assigned a CT value of −350 HU based on values obtained from the literature.^24^

### 2.C Volumetric and geometric analysis

The prostate, SVs, bladder, and rectum were delineated on the T2‐weighted images by a single physician in the Eclipse Treatment Planning System (Version 11.0,Varian Medical Systems, Palo Alto, CA). Boolean operations were used to generate the proximal 1 cm of the SVs (proxSVs) and the planning target volume (PTV) consisting of the prostate and proxSVs with a 1 cm expansion in all directions except posteriorly, which was expanded 0.6 cm. Overall, 170 evaluable contours were delineated in this study. For each volunteer, initial and intermediate T2‐weighted images of each subject and their corresponding contours were rigidly registered to the T2‐weighted image of the final time point (image with the largest bladder volume) using three parameter translation with mutual information as the cost function and nearest neighbor interpolation in FSL (FMRIB Software Library, Wellcome Center, Oxford, UK). This step ensured matching of the bony structures as the fixed components of the images across different time points as well as isolation of local effects such as possible movement (displacement) during the imaging period.

Next, for each time point, the contours of each organ were converted into a solid three‐dimensional volume using MATLAB (MATLAB R2016b, The MathWorks, Inc., Natick, MA, USA). The center of mass (COM) coordinates for each organ was calculated by finding the mean coordinates of all voxels of the volume along each major axis. The displacement of each organ was defined as the COM shift relative to the final time point.

### 2.D Dosimetric analysis

Using MR‐CAT images of the last time point (i.e., full bladder, which is consistent with our clinical practice), volumetric modulated arc radiotherapy (VMAT) plans were generated using two full arc beams with 6 MV photons. The treatment planning was designed to deliver 79.2 Gy to the PTV using RTOG 0815 dose constraints as a guideline.^25^ Next, plans were copied and recalculated to the synCTs of the other time points using fixed monitor units from the full bladder plans. After all the plans were created for each subject, an automated MATLAB program parsed the dose volume histograms (DVHs) in Eclipse and dosimetric data were tabulated for several dose metrics for the PTV and OARs according to QUANTEC recommended endpoints.^26^, ^27^, ^28^

### 2.E Statistical analysis

Repeated measures mixed models containing fixed (time points) and random (subjects) effects were used to assess the significance of changes of dose at different bladder states (initial, middle, and final) while using the multiple/repeated measures on the same subject. If the effect of the bladder states was significant (*P* < 0.05), pairwise comparisons of three time points using the overall mean square error (MSE) was calculated.

To investigate the associations between organ displacement and bladder and rectum volumes, multilevel modeling methods^29^ were used with the intercept coefficient as the random effect and the slopes (effect of changes of the bladder/rectum) as the fixed effect. Analysis was done both by considering the bladder and rectum volumes separately as well as in the same model. The testing level of significance was set at 0.05. All analyses were done using SAS version 9.4.

## 3 RESULTS

### 3.A Volumetric and geometric analysis

**Figure 2:**
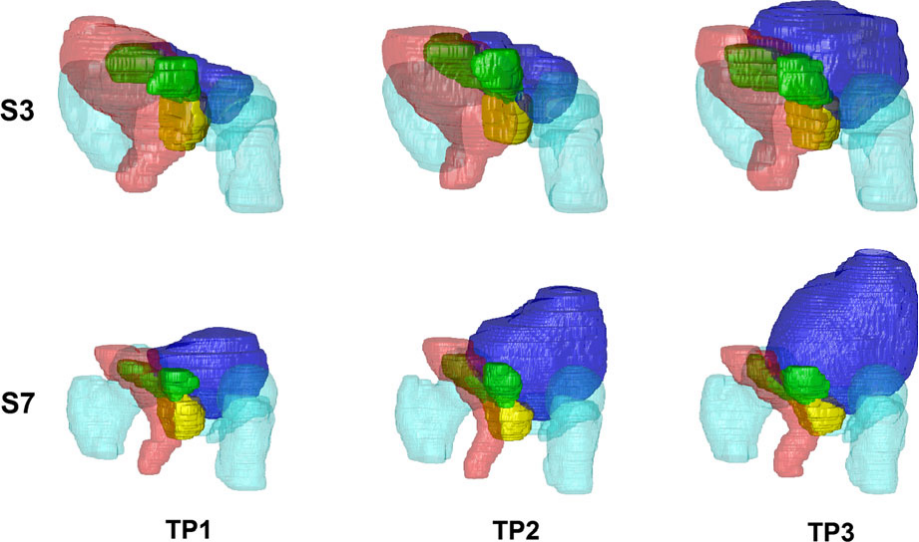
Three‐dimensional representation of the bladder (blue), rectum (red), prostate (yellow), seminal vesicles (green) and femoras/pubic bone (cyan) at three bladder filling time points (TP) for subject 3 (S3) and subject 7 (S7), who had the largest prostate displacement between states. The impact of bladder filling and rectal volume on the position of the prostate and seminal vesicles can be observed.

Subjects had an average bladder volume increase of 342.4 ± 284% between initial and final time points (137.26 ± 113.12 cc to 417.2 ± 262.1 cc) and the corresponding change in rectal volume was ‐6.9 ± 37.7% (102.8 ± 77.2 cc to 102.3 ± 57.9 cc). Figure 2 shows the 3D rendered volumes of the prostate and OARs for subjects 3 (largest rectal volume) and 7 (largest bladder volume), who also had the largest prostate vector displacements of ~6 mm. The dominant directions of the shift of the prostate between the bladder states were in the A‐P direction in both subjects. With reference to the COM coordinates of the prostate at the final time point, the A‐P movement of the prostate in subject 3 showed 5.92 and 5.61 mm displacement (anteriorly) at the second and first time points. The prostate displacement in subject 7 was 7.75 and 5.81 mm (posteriorly) in these two time points.

Table 1 summarizes the statistics for the displacement of the prostate and SVs between the full and empty bladder states. The prostate had a dominant vector displacement in the Anterior–Posterior (A‐P) direction, ranging from 1.3 to 5.6 mm across all subjects. Five of the nine subjects had a prostate displacement of higher than 2 mm along the A‐P direction (three subjects anteriorly and two subjects posteriorly). A significant negative association between the Superior–Inferior (S‐I) displacement of the prostate centroid with respect to volume changes in the bladder as well as a positive association with respect to changes in the rectal volume was observed. In addition, in the A‐P direction, there was a strong positive association between changes in the rectal volume and displacement of the prostate (*P* = 0.0001). The largest vector displacements were observed in the SVs (range of 3.1 to 9.3 mm). SVs for all subjects had >2 mm vector displacement, primarily in A‐P direction (range: 5.2 mm posterior to 7.8 mm anterior), with 7 of the 9 cases shifting in the anterior direction. In one subject, the S‐I displacement was dominant with 5.43 mm inferior shift. For the SVs, a strong association was observed between the displacement in the A‐P direction and the rectal volume changes, but no significant association was found with the bladder volume changes with either cardinal direction. The same results were also found when both the changes in bladder and rectum were considered in the same model. Physician delineations on the T2‐weighted images revealed that the overall prostate volume change was 1.0% ± 5.5% [range: (−6.4%, 10.3%)] between the empty and full bladder states.

**Table 1:** Centroid displacement of the prostate and seminal vesicles between initial and final time points for the cohort. Δx, Δy, and Δz represent displacement of the organ centroids in the LR, AP, and SI directions, respectively. The last two rows reflect the number of subjects that had an organ center of mass displacement of >2 mm along each axis or as the total vector displacement

	Prostate				SVs			
	Vector (mm)	Δx (mm)	Δy (mm)	Δz (mm)	Vector (mm)	Δx (mm)	Δy (mm)	Δz (mm)
Average	3.55	−0.29	1.35	0.61	5.50	−0.24	2.21	1.28
Stdev	1.87	0.62	3.50	2.26	1.67	2.01	3.54	2.87
Min	1.27	−1.19	−5.81	−4.05	3.09	−3.01	−5.20	−5.43
Max	6.10	0.89	5.61	3.36	9.30	3.31	7.81	4.80
# of patients > 2 mm shift	6	0	3	3	9	2	7	4
# of patients < −2 mm shift	N/A	0	2	1	N/A	1	1	1

Additional analyses were done to test whether the centroids for prostate and SVs change in the same way for changes in bladder and rectum volumes. For the changes in bladder and rectal volume, there was no significant difference observed between displacements in the Left–Right (L‐R) direction for prostate and SVs (*P* = 0.954 and *P* = 0.072, respectively). However, the differences between displacements in the SI and AP directions for prostate and SVs were significant (*P* = 0.0015 and *P* = 0.008, respectively, for changes in bladder volume and *P* = 0.028 and *P* = 0.028 for changes in the rectum volume).

### 3.B Dosimetric analysis

**Figure 3:**
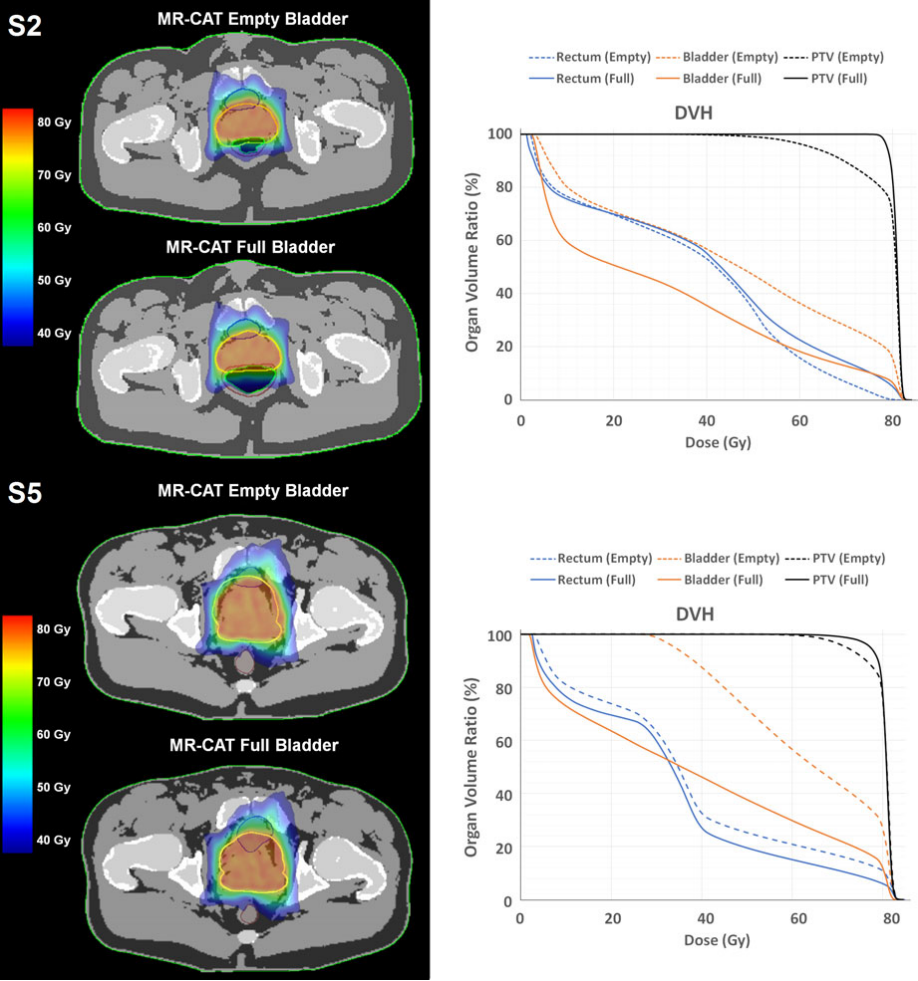
(Left) Synthetic computed tomography (CT) images with dose calculation results for the full and empty bladder states of subjects 2 and 5, with the initial plan optimized to the full bladder geometry. (Right) Dose volume histograms (DVHs) for the subjects at two bladder states. A reduction in planning target volume coverage can be observed due to the shift of the prostate from the initial planned location with changes in bladder and rectal conditions. As expected, increased bladder dose was also be observed due to smaller bladder volumes. This increase is more obvious in S5 where the bladder dose exceeds the recommended toxicity level.

Figure 3 highlights synthetic CTs for subjects 2 and 5 and their corresponding treatment plans that were optimized at the full bladder state and applied to the empty bladder geometry. Subject 2 had the highest percent increase of the rectal volume (52% or 71.5 cc) along with 56% increase in the bladder volume (125 cc) between the two time points. The prostate shifted posteriorly between the full to empty state due to the change in bladder and rectal volumes. DVH analysis revealed a 20.2% reduction in the D95% dose to the PTV and 22% increase of the D15% dose to the bladder (D15%(TP1) = 80.13 Gy) which is deemed not clinically acceptable.^28^ However, the mean dose to the rectum decreased by 11.98% (D15% (TP1) = 2.3 Gy).

Table 2 lists the minimum dose and D95% to the PTV as well as the D15%, D25%, and D35% to the bladder and rectum, as measured using the DVH of each of the nine subjects at the bladder full and bladder empty states. The tables show that the dose delivered to the PTV is reduced in every subject between the bladder full and bladder empty states and the dose delivered to the bladder is increased. Statistical analysis of the dose to the PTV shows that there is significant difference between the dose to the PTV between the full and empty bladder states. For change in bladder volumes, all of the associations with PTV dose measurements are positive and significant, except for maximum dose. Positive association indicates that dose to the PTV decreases as the bladder gets smaller. None of the associations between the changes in rectum volume with PTV dose measurements were significant. Similar findings were seen when both the changes in bladder and rectum were included in the statistical models.

**Table 2:** Dose volume histogram (DVH) metrics for the planning target volume (PTV), bladder and rectum for the nine subjects. For each subject, the top row represents the dose at the bladder empty state and the bottom row the dose at the bladder full state. Mean and standard deviation of the dose in the initial and final time points across all subjects has been calculated and the bottom row represents the significance of the difference between these doses in these two time points. Bold‐italized values indicate doses that are outside the accepted range

Patient	Status	PTV		Bladder			Rectum		
		Min D (Gy)	D95%(Gy)	D35%(Gy)	D25%(Gy)	D15%(Gy)	D35%(Gy)	D25%(Gy)	D15%(Gy)
S1	Empty	14.6	***66.9***	53.4	61.2	72.5	55.3	***68.60***	***80.53***
	Full	76.0	79.9	25.3	38.3	54.4	48.7	56.23	69.02
S2	Empty	31.9	***62.8***	61.6	73.5	***80.1***	49.7	53.76	60.78
	Full	73.2	78.8	40.6	51.3	65.5	51.0	57.75	69.05
S3	Empty	50.3	***69.7***	60.4	67.1	75.7	40.9	49.78	63.89
	Full	73.8	78.7	25.3	39.9	55.2	42.0	49.16	59.96
S4	Empty	28.4	***68.9***	36.7	49.8	67.8	38.0	43.61	59.13
	Full	50.8	76.9	18.2	27.4	50.4	44.4	61.90	***77.74***
S5	Empty	47.3	***72.1***	***76.8***	***80.3***	81.3	38.9	50.48	73.63
	Full	56.9	77.2	53.3	66.5	79.0	37.4	41.45	60.22
S6	Empty	50.3	74.6	74.7	***80.4***	***81.6***	41.5	47.11	57.77
	Full	69.5	77.6	47.9	51.0	72.8	46.9	55.38	69.33
S7	Empty	30.3	***62.9***	34.2	44.4	61.1	44.5	54.50	64.03
	Full	74.3	79.3	3.4	5.9	24.4	44.0	55.99	72.37
S8	Empty	58.7	***71.2***	31.2	38.4	47.1	43.4	51.15	63.48
	Full	74.1	79.0	4.1	9.1	32.3	37.1	46.22	56.26
S9	Empty	54.6	75.5	***78.6***	***80.4***	***81.0***	52.9	61.52	73.62
	Full	72.9	78.7	30.8	41.5	57.1	53.9	63.06	74.48
Mean ± SD	Empty	11.8 ± 11.0	69.4 ± 4.3	56.4 ± 17.7	63.9 ± 15.5	72.0 ± 11.0	45.0 ± 5.9	53.39 ± 7.15	66.32 ± 7.33
	Full	1.6 ± 1.2	78.5 ± 1.0	27.7 ± 17.6	36.8 ± 19.8	54.6 ± 17.6	45.0 ± 5.4	54.13 ± 6.75	67.60 ± 6.84
P value		0.0003	0.002	<0.0001	<0.0001	<0.0001	0.9919	0.9158	0.9743

## 4 DISCUSSION

In this work, we did a systematic study of effects of bladder and rectal volumes on displacement of the prostate and surrounding organs as well as the impact of this displacement on the delivered dose to the PTV and (OARs). The bladder filling experiment was designed such that it made possible to model extreme ranges of the bladder volume and to observe the range of its effects.

Although previous studies seeking to find an optimal bladder and rectal state^30^, ^31^ for prostate radiotherapy have not found significant differences in the intra‐fractional prostate displacement between plans that were designed for patients with full and empty bladders, they have not investigated displacement and change in the dose to the prostate between extreme bladder states. Our results showed that changes in the bladder volume can lead to large, systematic displacements in the prostate and SVs. The major displacements are observed in the A‐P and S‐I directions in both organs. While the prostate is shifted anteriorly in most cases as the bladder volume is reduced, in some cases, posterior shift is observed. These findings match the results of a recent study based on analysis of CBCT and four‐dimensional (4D) trans‐perineal ultrasound (4D TPUS) measurements of 60 patients that showed intra‐fractional motion of the prostate in the A‐P and S‐I directions.^32^ In this study, reduction of A‐P motion of the prostate was observed when the planned bladder volume was greater than 200 ml. Also, when the daily bladder volume was within the third quartiles of the planned CT volumes, the A‐P and S‐I intra‐fraction displacement of the prostate was reduced.

The major contributor to vector displacements of the prostate and SVs is the change in bladder volume; however, rectal status/volume can also contribute to the range of displacements of these organs, which can be dominant along different axes. This can be observed in subject 7 (Fig. 2) where the displacement of the prostate can be related to changes in the rectal volume. In this subject, the rectal volume reduces in the second time point and increases in the first time point. Relative to the third time point, the prostate initially moves posteriorly at the second time point and anteriorly at the first time point. This matches the results of our statistical analysis which showed that the rectum volume is the main effector of displacement of the prostate in the A‐P direction. It should be noted that in our study, considering the change of rectal gas volume between different measurements in some subjects, variations of susceptibility‐based distortions might affect the measured displacement of the organs near the rectum. However, previous measurements done across three magnetic field strengths (1.0, 1.5, and 3.0 T) showed that only 1.4% and 1.9% of all voxels in the Prostate and SVs were distorted by greater than 0.5 mm^33^; therefore we do not expect the susceptibility‐based distortions to adversely impact our results.

A previously published CT‐based treatment planning study evaluated the dosimetric impact of full and empty bladders.^18^ The present study builds upon this previous work by incorporating MRI across 3–5 time points per subject. MRI has been shown to enable more accurate and more consistent delineations.^34^ Further, Moiseenko et al. found that bladder filling status had limited dosimetric impact on the prostate and rectal doses; however at that time, treatment planning was conducted using a four‐field box technique.^18^ Our work implements much more conformal treatment planning using arc therapy which showed that when the bladder volume changes from full to empty, PTV coverage was adversely affected. Finally, while CT is the gold standard for treatment planning, the present work is the first to evaluate the performance and dosimetric impact of synthetic CT across varied subject anatomies.

Previous studies have shown that using different table‐top configurations used in MRI (i.e., flat and curved couches) may lead to changes in the relative location of pelvic organs.^35^ When performing MRI scans with the patient in treatment position (i.e., using a flat tabletop similar to the treatment couch vs. a curved diagnostic couch), more accurate rigid registrations between MR images and CT images for prostate RT planning has been observed.^36^ It has also been shown that during MR‐SIM, the weight of the flexible anterior body coils may contribute to changes in the position of pelvic organs.^34^ In this study, serial imaging data were acquired using a single setup for each subject (i.e., subjects did not leave the MR table‐top during scanning and no change in the body coil placements were made during acquisition). By conducting a within‐subject analysis, the impact of the table‐top selection and body coils may be considered negligible within a particular subject. However, in clinical practice, acquiring MRI data in the treatment position improves agreement between the anatomy at time of treatment planning and during treatment.^34^

One limitation in the current work is that only nine healthy volunteers were evaluated. Nevertheless, this yielded 34 overall sample points and enabled statistical comparisons to be made for both geometry and dosimetry across the target and OARs. Another limitation of this work is that healthy volunteers were evaluated that may not be representative of the average prostate cancer population. However, because this work included empty to very full bladder volumes for each subject, we expect that the results, even at intermediate states, will extend to the prostate cancer population. Another limitation of this work is that because MR‐CATs were derived from healthy volunteers, no corresponding CTs were available for quantitative MR‐CAT evaluation. However, a recent study illustrated a good agreement between MR‐CAT datasets and their corresponding CTs with a low MAE in a patient cohort.^12^

In the simulation that we performed, by evaluating the dose delivered to the organs at the bladder empty state using the treatment plan optimized for the dose delivered to the PTV at the bladder full state, we observed that reduction of the bladder size from the full state can lead to decreased delivered doses to the PTV and increased dose to the bladder. Our study did not reveal any significant change in the dose delivered to the rectum. This simulation further revealed that due to the shift of the PTV, the dose to the bladder may exceed the maximum recommended dose to the bladder. Our results confirm the results of another study by Chen et al. which reported that increases in the bladder volume lead to reduction of the dose to the prostate.^17^ Using CBCT images of 19 subjects, they found that a 10% increase in bladder volume leads to 5.6% reduction of mean dose to the prostate. They did not find significant variations of the rectal volume. These findings contradict a previous report that although confirming displacement of the prostate in the A‐P direction after voiding the bladder, found no correlation between prostate shifts with bladder and rectal volume.^18^ These results may be due to that study only evaluating two bladder states (full and empty), and imaging/contouring was done using CT images. Also, the reference for prostate motion was based on external fiducial markers.

Considering that contouring the organs might introduce some uncertainty in their size and position, we eliminated the possible inter‐observer error by having only one physician delineate the organs in all subjects, based on the protocol guidelines of RTOG 0815.^25^ This minimized the differences between the organ contours for each subject at different time points to ensure isolation of volume differences and their effects on the organs.

## 5 CONCLUSION

Variations in the bladder volume can lead to the displacement of the prostate which can negatively impact the dose delivered to the PTV and the bladder. These results show the importance of proper preparation of patients both for treatment and also during imaging sessions, especially when imaging requires longer scan times such as MR protocols.

## CONFLICT OF INTEREST

Henry Ford Health System holds research agreements with Philips Medical Systems. SW and SR are Clinical Scientists at Philips Healthcare who made contributions to the data collection, experimental design, and analysis in this work.
